# Efficacy of a Food Safety Comic Book on Knowledge and Self-Reported Behavior for Persons Living with AIDS

**DOI:** 10.1371/journal.pone.0072874

**Published:** 2013-10-04

**Authors:** Mark S. Dworkin, Caryn E. Peterson, Weihua Gao, Angel Mayor, Robert Hunter, Edna Negron, Alison Fleury, C. Lynn Besch

**Affiliations:** 1 Division of Epidemiology and Biostatistics, University of Illinois at Chicago School of Public Health, Chicago, Illinois, United States of America; 2 Center for Clinical and Translational Science, University of Illinois at Chicago, Chicago, Illinois, United States of America; 3 Retrovirus Research Center, Internal Medicine Department, Universidad Central del Caribe, School of Medicine, Hospital Universitario Ramón Ruiz Arnau, Santa Juanita, Bayamón, Puerto Rico, United States of America; 4 Food Science and Technology Program, University of Puerto Rico-Mayagüez, Mayagüez, Puerto Rico, United States of America; 5 Department of Medicine, Louisiana State University Health Sciences Center, New Orleans, Louisiana, United States of America; Tulane School of Public Health and Tropical Medicine, United States of America

## Abstract

**Introduction:**

Persons living with AIDS are highly vulnerable to foodborne enteric infections with the potential for substantial morbidity and mortality. Educational materials about foodborne enteric infections intended for this immunocompromised population have not been assessed for their efficacy in improving knowledge or encouraging behavior change.

**Methods/Results:**

AIDS patients in four healthcare facilities in Chicago, New Orleans, and Puerto Rico were recruited using fliers and word of mouth to healthcare providers. Those who contacted research staff were interviewed to determine food safety knowledge gaps and risky behaviors. A food safety educational comic book that targeted knowledge gaps was created, piloted, and provided to these patients who were instructed to read it and return at least 2 weeks later for a follow-up interview. The overall food safety score was determined by the number of the 26 knowledge/belief/behavior questions from the survey answered correctly. Among 150 patients who participated in both the baseline and follow-up questionnaire, the intervention resulted in a substantial increase in the food safety score (baseline 59%, post-intervention 81%, p<0.001). The intervention produced a significant increase in all the food safety knowledge, belief, and behavior items that comprised the food safety score. Many of these increases were from baseline knowledge below 80 percent to well above 90%. Most (85%) of the patients stated they made a change to their behavior since receiving the educational booklet.

**Conclusion:**

This comic book format intervention to educate persons living with AIDS was highly effective. Future studies should examine to what extent long-term behavioral changes result.

## Introduction

Persons living with AIDS who have CD4 T-lymphocyte counts < 200 cells/mm^3^ are highly vulnerable to foodborne enteric infections with the potential for substantial morbidity and mortality. Compared to the general population, the incidence rates of gram-negative bacterial enteric infections (such as *Salmonella* and *Campylobacter* ) are 20 to 100-fold higher in those with HIV infection [1]. Although highly active antiretroviral therapy (HAART) is frequently successful at controlling viral reproduction, some patients spend months and sometimes years with a low CD4 cell count despite control of their viral load [2,3]. The morbidity and mortality experienced by persons living with AIDS may also be greater than that experienced by persons without immune system impairment as evidenced by cerebral toxoplasmosis, recurrent *Salmonella* bacteremia, and listeriosis [4–10].

Food safety educational material does exist for AIDS patients [11–14]. However, such materials have not been assessed for their efficacy in improving knowledge or encouraging behavior change, receptivity of the target population, and the extent of availability. They also have certain limitations worth considering. An FDA brochure initially available in 1992 entitled Eating Defensively: Food Safety Advice For Persons With AIDS includes paragraphs with advice on shopping for food, the home kitchen, eating out, and traveling abroad [11]. However, the brochure includes pathogen specific clinical information which may be more detail than needed for the target population, lacks illustrations, and requires a computer and Internet to access since attempts to acquire a paper copy were not fruitful (including calling the telephone number provided on the Internet page). The USDA produced a 19-page brochure in English and Spanish called Food Safety for People with HIV/AIDS: A need-to-know guide for those who have been diagnosed with HIV/AIDS [12,13] derived in part from focus groups with HIV-infected patients and healthcare providers in Washington, Ohio, and Colorado [15]. This brochure includes many detailed pages of information including recommended internal cooking temperatures for common foods, a table of types of foods with information on higher versus lower risk preparations, and general information on what to do when you suspect you have a foodborne illness. However its length and quantity of information may be limitations. It also may be viewed, in part, as technical because it includes a pathogen by pathogen two-page table that appears to be designed for self-diagnosis, a large amount of text, and some language that may be beyond the knowledge of many in the target population, such as “diarrhea is more prevalent in adults,” and open and closed dating. The CDC produced a brochure entitled, Safe Food and Water in English and Spanish [14]. Its format is question and brief answer. The questions are of a personal nature such as, “Can I eat eggs if I have HIV?” and “What should I do when shopping for food?” Unlike the very thorough and more recently created USDA brochure, the CDC brochure has generic rather than educational illustrations. The FDA, CDC and USDA brochures also provide telephone numbers to government agency food safety information hotlines. None of these brochures have been evaluated for receptivity, efficacy, and extent of availability to the target population.

We have studied and reported substantial knowledge gaps in persons living with AIDS in Chicago, New Orleans, and Bayamón, Puerto Rico [16]. In addition to lack of basic knowledge about foods and food preparation practices that may place them at relatively high risk for transmission of pathogens, this survey also revealed that many patients lacked awareness of the potential clinical consequences of these foodborne infections. Without such awareness, patients might be less likely to follow recommended food safety practices. Therefore, we created an English and Spanish language food safety educational comic book that targeted knowledge gaps identified in this population and included information raising their awareness of the clinical consequences of some of these foodborne diseases. The objectives of this study were to determine the efficacy of this food safety comic book to improve knowledge and to determine self-reported behavior changes that followed exposure to this educational intervention.

## Methods

### Educational Intervention

A 15-minute questionnaire was developed to obtain information about the knowledge and behaviors of patients with AIDS that had a self-reported CD4 T lymphocyte cell count below 200 cells/mL or <14% within 3 months before the interview. The design of this questionnaire and results from its use have been previously reported [16]. AIDS patients were interviewed face-to-face at the Ruth M. Rothstein CORE Center of the John R Stroger Jr. Hospital and the University of Illinois at Chicago Family Center for Immune Deficiencies and Infectious Diseases in Chicago, the Louisiana State University Health Sciences Center HIV Outpatient Clinic in New Orleans, and the Retrovirus Research Center, Universidad Central del Caribe School of Medicine and University Hospital Ramón Ruiz Arnau in Bayamón, Puerto Rico.

Patients were recruited using fliers placed in patient care rooms and word of mouth to healthcare providers during 2010 through March 2012. Twenty dollars compensation was offered for participation in the baseline and follow-up interviews. Patients were excluded if they could not consent for themselves, did not speak either English or Spanish fluently, were prisoners, were younger than 18 years of age in Chicago and New Orleans or younger than 21 years in Puerto Rico, or had a CD4 T-lymphocyte cell count >200 cells/mL and at least 14%. A signed consent form was obtained from each participant and confidentiality was assured. Approval from the Institutional Review Board for the Protection of Human Subjects was obtained from each of the four participating sites before the initiation of the study. Patient’s provided their contact information (usually telephone number) to reach them for the remaining portion of the study.

**Figure 1:**
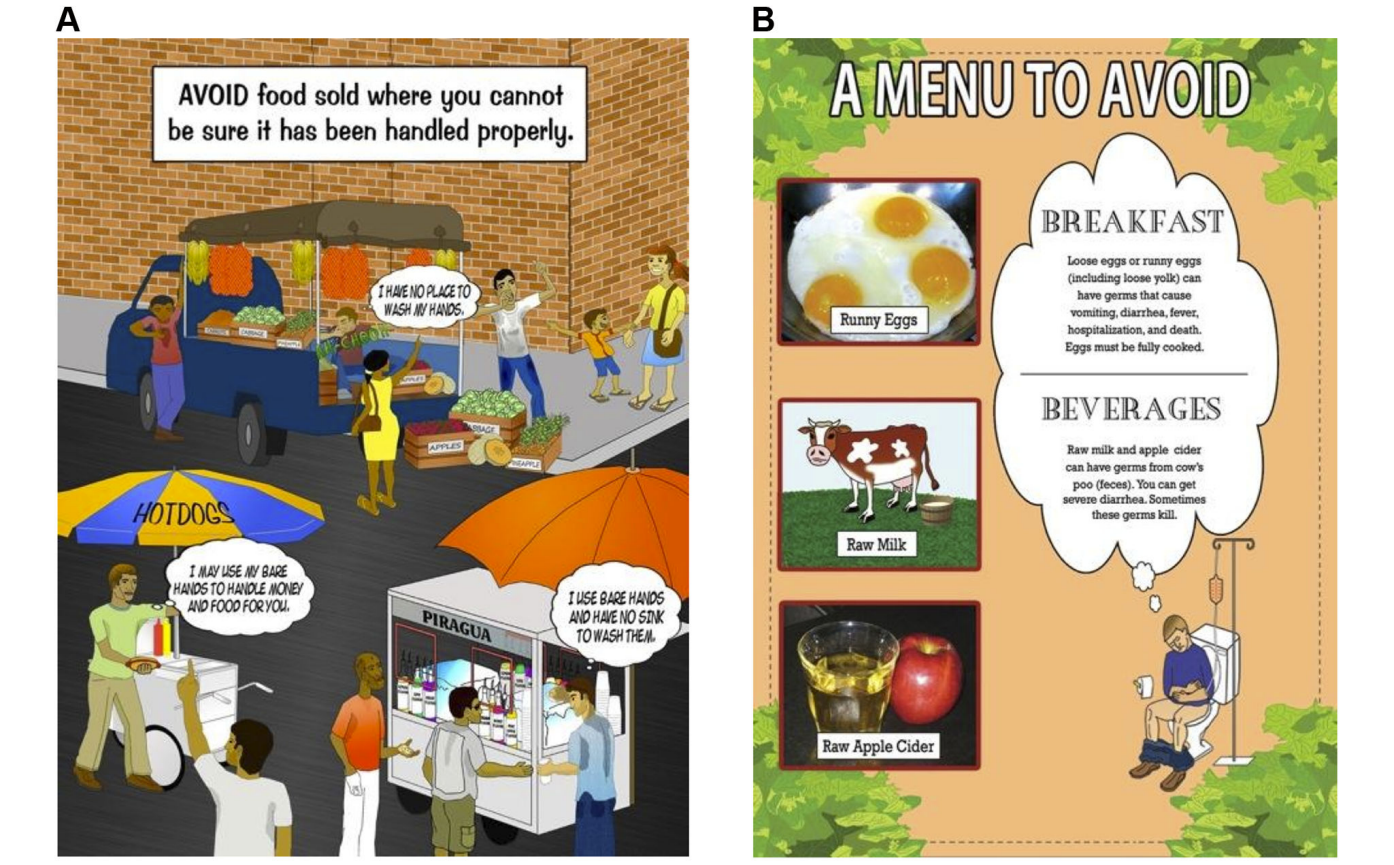
A page from the food safety comic book for AIDS patients (A) explaining the rationale for avoidance of food sold by street vendors including truck produce, hot dog, and piragua (shaved flavored ice) vendors. (B) One of four pages from the food safety comic book for AIDS patients that presented a menu of foods to avoid and potential health consequences that could occur if these foods are eaten.

The food safety comic book, entitled “Food Safety: If You Eat, Read This,” was developed by one of the authors (MSD) in collaboration with an artist, with input from collaborators at each of the participating sites and then edited further after review by HIV patient focus groups of five persons in each city. Each focus group recipient received $50 as compensation for their time. The rationale for a comic book format was to visually attract, illustrate graphically, and use storytelling as methods of generating interest while educating. Comic books and similarly photonovellas have been successfully used to teach health-related prevention information in other settings [17–19]. With abundant illustrations, the 20 page (16 plus four cover pages) comic book emphasized basic health literacy including the definition of pasteurization, the steps of hand hygiene, the definition and rationale for hepatitis A vaccination, and the importance of adherence to antiretroviral medication to preserve or improve immune system health. Other topics included best food handling and cooking practices, circumstances to avoid (such as conditions where food is sold in the absence of good hygiene), and food preparations to avoid. Health consequences of foodborne disease were also emphasized through text and graphic illustration (Figure 1a and 1b). The comic book was distributed to patients beginning approximately 9 months after taking the baseline survey due to the time needed to create and edit the comic book. Receipt of the comic book at their healthcare clinic was accompanied by $10 compensation for their time and to offset transportation cost. Each recipient was instructed to read it but no explanation of content was provided. A follow-up questionnaire was administered face-to-face at least 2 weeks after that and as soon as the patient could be conveniently scheduled.

Only patients who received the comic book and participated in both the pre-intervention and follow-up questionnaires were included in the efficacy analysis. Baseline characteristics for those who participated in the entire study versus those who were lost after baseline interview were compared and differences were tested using t-test and Chi-square test statistics. The time span (in days) between the intervention date and follow-up interview was calculated by subtracting the date of follow-up interview from the date of intervention. Questions that were common to the pre-intervention and follow-up surveys were tabulated for the intervention group to determine changes over the two study phases. Differences were tested using paired t-test and McNemar for continuous and categorical variables, respectively. A variable called the food safety score was create based on the number of knowledge, belief, and behavior questions relevant to foodborne illness risk that were answered correctly. A response of “Not sure” was categorized as an incorrect response because it represented a lack of knowledge. The overall food safety score was determined by the number of the 26 knowledge/belief/behavior questions from the survey answered correctly out of all of such questions. In the case of belief and behavior questions, responses that would place the patient at lower risk for foodborne disease were considered correct. For the question that asked about the safest option for handling raw fruits (watermelon, cantaloupe, papaya, and grapes), a food safety point was awarded if they did not eat any of these fruits and therefore were unlikely to put themselves at risk from mishandling any of them or they handled all of those safely. A point was not awarded if they did eat at least one of these fruits and did not handle it in the safest way. We calculated a pre-intervention FSS and a follow-up FSS and compared the scores for the overall study population and stratified by site using paired t tests. Statistical analysis was performed using the statistical package SAS (version 9.2, SAS institute, Cary, North Carolina).

### Survey of Outpatient HIV Primary Care Providers

We also performed a survey of outpatient facilities where patients with AIDS receive care in Chicago, New Orleans, and the island of Puerto Rico. Using several Internet search engines including the American Academy of HIV Medicine and the telephone book listing of infectious disease physicians, a list of providers of HIV care was generated for the two cities and the island. This list was then reviewed by a local HIV specialist collaborator to identify any missing sites. Each of the sites was telephoned and those that provided HIV primary care to at least 5 patients were asked if they had any educational materials that taught about food safety prevention. If they responded yes, the names of those materials were recorded. They were also asked if their facility had a dietitian. The educational comic book was mailed to each of these sites at the conclusion of the study.

## Results

### Educational Intervention

The baseline survey was completed by 298 patients. One hundred and fifty of these patients participated in the intervention (receipt of the comic book and follow-up survey). Compared to those who completed only a baseline survey, those who completed the entire study had a higher proportion of Hispanic Whites and a lower proportion of African Americans (35% intervention versus 19% only baseline and 47% intervention versus 64% only baseline, respectively, p value 0.009). Concerning their educational background, those who remained in the study were more often at the extremes of education (11%, 57%, and 32% with <high school, high school, and college or higher education for the baseline only group versus 19%, 45%, and 46% for those remaining in the study, p value 0.05). Those who were lost after the baseline versus those who participated in the entire study were not different based on age, sex, or baseline food safety score (64% versus 63%). The median time span between receipt of the comic book and follow-up interview was 16.5 days with a range of 1 to 274 days (25^th^ percentile 7 days, 75^th^ percentile 35 days). The intervention resulted in a substantial increase in the FSS (baseline food safety score 59%, post-intervention food safety score 81%, p<0.001).

The comic book intervention produced a significant increase in all of the food safety knowledge, belief, and behavior items that comprised the food safety score (Table 1). Many of these increases were from baseline knowledge below 80 percent to well above 90% such as concerning the potential danger of eating eggs that are not fully cooked, store bought hot dogs that have not been cooked, and food that is past its expiration date even if it does not smell or look bad. In addition, the intervention raised knowledge that certain foods have been associated with illness that can hospitalize or cause death in immunosuppressed persons. At baseline, there was little recognition of the potential for foodborne disease from food items often causing outbreaks that were reported widely in the news in the past two decades such as sprouts and raw green leafy vegetables (below 30%). However, such recognition improved after the intervention, rising to the 60% to 70% range.

**Table 1:** Percent distribution of correct responses to food safety questions and comparison of changes between the pre-intervention and follow-up phases.

Question (correct or preferred response)	% Correct Response Pre-intervention* N = 150	% Correct Response N = 150	P Value
Risky Food Consumption Knowledge			
“Is it okay to eat ordinary eggs (not pasteurized eggs) served loose or runny such as soft-scrambled or sunny-side up?” (*Not okay*)	63	94	<0.0001
“Is it okay to eat store-bought hot dogs (such as those that come in a plastic package) without heating them first?” (*Not okay*)	77	98	<0.0001
“Is it okay to eat food that requires refrigeration past its expiration date if it does not look or smell bad?” (*Not okay*)	79	97	<0.0001
“Is it okay to eat a pork chop that is rare (not completely cooked in the center)?” (*Not okay*)	93	97	0.07
“In a person with AIDS, eating which of these following foods may get germs inside your body that could cause hospitalization and possibly death?” (*All of these foods are risky for persons with AIDS, especially with very low CD4 T-lymphocyte counts*)			
Raw carrots	23	66	<0.0001
Bean sprouts	33	63	<0.0001
Lettuce	28	63	<0.0001
Raw spinach	29	74	<0.0001
Cooked eggs with loose yolks	36	89	<0.0001
Unpasteurized apple cider	49	86	<0.0001
Unpasteurized cheese	57	91	<0.0001
Cold cuts like salami or bologna	53	76	<0.0001
Medium-rare lamb	77	93	0.0001
Hamburger served medium-rare	83	94	0.001
Pork that is not completely cooked	91	97	0.02
Fried chicken that is pink in the center	88	95	0.03
Risky Food Handling Behavior			
“Which method of handling watermelon, cantaloupe, papaya, and/or grapes best applies to you?” (*I wash the fruit with plain water before cutting or eating it*)**	64	75	0.05
“Imagine you are making a salad and preparing to cook chicken for the same meal. If a few drops of raw chicken juice are splashed on to the salad, what should be done with the salad?” (*Throw away the salad*)	35	55	<0.0001
Food Handling Knowledge			
“Is it okay to thaw frozen ground meat (hamburger) on the counter?” (*Not okay*)	70	88	<0.0001
“Imagine this situation: raw chicken is cut up on a cutting board. Next the cutting board is rinsed with warm water, and then fresh fruit is cut on the cutting board.” (*Not okay*)	64	75	0.02
“Cooked and ready-to-eat foods (like salad) should be kept separated from raw meat, poultry, seafood, and their juices.” (*Yes*)	89	96	0.02
“Packaged meat, poultry, or fish should be wrapped in plastic bags to prevent their juices from dripping onto other groceries or onto each other.” (*True*)	91	98	0.01
Time and Temperature Knowledge/Beliefs			
“Do you believe that leftovers (like cooked chicken, other cooked meat, or other cooked dishes) should be reheated before eating?” (*Yes*)	86	95	0.001
“To what temperature should leftovers (like cooked chicken, other cooked meat, or other cooked dishes) be reheated before eating?” (*>140°F*)	9	30	<0.0001
"Do persons with AIDS need to heat lunch meats even if they are labeled “fully cooked, ready to eat?” (*Yes*)	55	85	<0.0001
“At what temperature should a refrigerator be kept at in order to make sure that food is kept at a safe temperature to prevent germs from growing?” (*40°F*)	<1	42	<0.0001

Many patients recognized that raw meat, such as pork or chicken, could make them sick if not completely cooked and that cooked and ready-to-eat foods should be kept separated from raw meat, poultry, seafood, and their juices. However, questions that asked about actual cross contamination-related behavior reveal that while the intervention raised awareness to avoid certain behaviors, there were still many patients who might still place themselves at risk. For example, although there was improvement in recognition that salad contaminated with raw meat juice should be thrown away, almost half of the patients responded that they might still consume it. Similarly, 30% of patients post-intervention did not respond that cutting fresh fruit on a cutting board that had been used to cut raw chicken and was only rinsed in between with warm water was not an acceptable practice.

Specific knowledge related to temperature was poor. Although nearly all the patients believed that leftovers should be reheated, the majority of the patients did not know to what temperature the leftovers should reach even after this was taught in the comic book. Also, although knowledge of appropriate refrigerator temperature rose substantially with the intervention, still nearly 60% of the patients did not know.

Most (85%) of the patients reported changing their behavior after receiving the educational booklet. This was highest in Chicago (94%) and lower in Puerto Rico (84%) and New Orleans (74%). Examples of behavior changes included the following. “Make sure my fried chicken and pork is cooked well. Reheat my leftovers.” “I heat my lunch meat and stopped eating runny eggs.” “I cook my hot dogs.” “Don’t buy food that can’t be washed like a street vendor.” “Wash the fruit first, like watermelon.” “If raw meat juice spills onto other food I won’t use that food. Scrub my fruit and vegetables.” “Cooking wieners and cold cuts, washing cutting board between chicken and fruits.” “Cut down on sushi.”

### Survey of Outpatient HIV Primary Care Providers

Among 96, 16, and 35 outpatient facilities identified in Chicago, New Orleans, and Puerto Rico, respectively, 43, 8, and 15 were contacted successfully and willing to respond to the survey. All of these (except three in Chicago) had at least five HIV patients. Only 2 (5%), 1 (13%), and 4 (27%) clinics, respectively, had food safety materials available for their patients. Five of these were produced by the FDA, one by the USDA, and one by the Channing Bete Company®. A dietitian was available at 7 (18%), 4 (50%), and 8 (53%) of these facilities.

## Discussion

Patients with AIDS are an important population to target for food safety education due to their compromised immune systems, low baseline food safety knowledge and potentially risky food safety behaviors [16]. We chose to focus on persons living with AIDS who had CD4 T-lymphocyte counts fewer than 200 cells/mL or percentage below 14 because these measurements represent immunological AIDS with its accompanying heightened risk for opportunistic infections. Given their current immunosuppression, they might receive the greatest benefit from education about prevention of foodborne disease. This is the first study of an intervention directed at raising food safety knowledge in patients with AIDS. The results demonstrate significant efficacy of the intervention comic book. Moreover, this study suggests that educational material designed to target food safety knowledge gaps and behaviors delivered in a colorful and illustrated story-telling format is acceptable and results in self-reported behavior change for many of the study population.

Knowledge concerning the importance of cooking eggs completely to prevent foodborne illness was among those demonstrated to substantially improve. Knowledge related to risk from eggs served loose or runny rose from 63% to 94% and that cooked eggs with loose yolks can cause hospitalization and possibly death rose from 49% to 86%. Large increases in knowledge were also observed related to foods that have been highly publicized for contaminating food that led to national outbreaks of food poisoning from pathogens including *Salmonella* , *Listeria* , and *E. coli* [20–22]. However, while all the items comprising the food safety score increased significantly, several issues remained poorly recognized by the study population after the intervention. These included recognizing the importance of discarding food intended to be consumed raw that had been contaminated from raw meat or poultry, knowledge of to what temperature leftovers should be reheated and knowledge of how cold to keep a refrigerator. Future study or educational efforts should consider these issues both regarding how to best instruct this population and their receptivity to acting on reducing their risk by obtaining and using refrigerator and meat thermometers.

The comic book evaluated in this educational intervention was unique compared to the FDA, USDA, and CDC brochures. One difference included the design of the cover because focus group input recommended that it make no mention of HIV or AIDS and not even display the AIDS red ribbon symbol. This was due to concern over stigma since if someone else recognized that the information was for HIV-infected persons then they may assume the reader is HIV positive. Terms such as pasteurized were prominently and simply defined and many illustrations were designed to demonstrate correct and incorrect behavior by placing a large red X over the image in an attempt to reach persons of low literacy. Full pages were devoted to minimal words and prominent illustration of key basic behavioral advice on proper hand hygiene and when hand hygiene is most indicated. Higher risk scenarios for transmission of disease from others in settings where their personal hygiene might be compromised (produce truck markets and street vendors) were illustrated. Awareness of personal risk was presented with straightforward facts such as “Some fruits and vegetables such as watermelon, strawberries, and spinach can still have soil and feces on them after being purchased. There are germs in soil and in bird and animal feces that can make people sick and even die.” Steps to take to reduce risk from such foods accompanied such provocative language. Two large illustrations (one within and one full-page on the back cover) emphasized the importance of medication adherence as “The best recipe for your health,” since antiretroviral adherence is the best method of reducing increased risk for foodborne disease that accompanies the loss of CD4 T-lymphocytes.

While education on foodborne disease prevention is important for persons with AIDS, our HIV healthcare provider survey revealed that few of these providers had any of the brochures mentioned above. Potential reasons for their lack of availability include provider unawareness and low prioritization of prevention of foodborne disease in this population. Regardless, these data quantify the lack of availability and demonstrate that these educational materials are not likely reaching their target audience.

A limitation of this study is that a response of “Not sure” was categorized as an incorrect because it represented a lack of knowledge. That doesn’t necessarily mean that people who responded “Not sure” were performing the unsafe behavior related to it. Therefore, the results we report may overestimate risk associated with lack of knowledge. This study did not include a control group. Although it is possible that knowledge could have increased due to factors other than the comic book, this is unlikely. The study measured self-reported rather than observed behavior. Therefore, it might overestimate reported behavioral change. The use of financial compensation for patient’s time could have been viewed by some patients as an incentive and they might not have read the comic book if it was passively placed in a brochure rack. Another limitation of the study was loss to follow-up. The study methods required a relatively long gap between the baseline survey and the follow-up (approximately 9 months). That time gap allowed for design of the educational materials in direct response to the knowledge gaps identified in the baseline survey. However, given the mobility of this patient population, it also allowed for many to no longer be available as many telephone numbers provided by patients no longer worked. Future studies would benefit from a modified design that obtained knowledge gap data by focus groups and then created the educational material. This way, the baseline and follow-up surveys could then be performed much closer in time since educational material would be created prior to baseline survey administration. Finally, a limitation was the sample size, which was not large enough to allow for evaluation of the intervention by subgroups.

Improving food safety and reducing foodborne illness is a Healthy People 2020 goal [23]. Among the food safety objectives are to increase the proportion of consumers who follow key food safety practices and to reduce foodborne infections caused by *Salmonella* , *Campylobacter* , *Yersinia* , and *Vibrio* species, Shiga toxin-producing *Escherichia coli* O157, and *Listeria monocytogenes*. Greater attention is needed to this basic health issue since AIDS patients are a highly vulnerable population for foodborne disease due to their immunocompromised condition, their lack of food safety knowledge [16,24], and their higher risk for morbidity and mortality [5–7]. Food safety educational materials should be more broadly available and incorporated into prevention counseling of AIDS patients along with other routine prevention messages such as those directed at immunization, medication adherence, and avoidance of STDs. This study demonstrated that AIDS patients generally enthusiastically receive and learn from food safety material. In addition to wanting to avoid illness, they likely find some food safety behavior easier to change than sexual or substance abuse behaviors. We recommend greater availability of food safety educational materials to persons living with AIDS.
